# Water pipe smoking and stroke: A systematic review and meta‐analysis

**DOI:** 10.1002/brb3.3357

**Published:** 2024-01-02

**Authors:** Mehrdad Bagherpour‐Kalo, Michael E Jones, Parvaneh Darabi, Mostafa Hosseini

**Affiliations:** ^1^ Department of Epidemiology and Biostatistics School of Public Health, Tehran University of Medical Sciences Tehran Iran; ^2^ Division of Genetics and Epidemiology The Institute of Cancer Research London UK; ^3^ Department of Biostatistics School of Public Health Iran University of Medical Sciences Tehran Iran

**Keywords:** hookah, meta‐analysis, stroke, systematic review, water pipe

## Abstract

**Objective:**

Despite the damaging effects of water pipe on physical health, there is little information about the potential harmful effects of this tobacco on stroke. This study aims to investigate the relationship between water pipe smoking and stroke.

**Method:**

A systematic review was conducted including Ovid SP, Embase, Pubmed, Web of Science, Scopus, and Google Scholar databases with focus on cohort, case–control, and cross‐sectional studies. We reviewed all studies reporting on water pipe smoking and stroke. The funnel plot and the Egger regression test were used to assess publication bias.

**Results:**

In the four eligible studies, there were a total of 2759 participants that 555 patients had at least once experienced stroke. Meta‐analysis revealed positive association between water pipe smoking and stroke with pooled adjusted OR 2.79 (95% CI: 1.74–3.84; $I^{2}\;=\;\;0,\;p\;\;=\;\;.741$) and the funnel plot shows asymmetry publication bias.

**Conclusions:**

We found a higher effect of water pipe smoking on stroke compared to cigarette smoking and concluded that water pipe increases the risk of stroke by 2.79. Hence, because most of the water pipe consumer society is young, especially women, policies and decisions need to be taken to control the supply of this tobacco to the market and more provide education on the health problem of water pipe smoking.

**Implications:**

This study provides a higher effect of water pipe smoking on stroke. Physicians and researchers who intend to study in the field of stroke should better examine the effects of water pipe (including time of use, dose–response, long‐term effects, and risk factors) on stroke.

## Graphical abstract

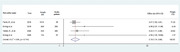

## 1 INTRODUCTION

Stroke, the second leading cause of mortality worldwide, exerts a profound impact on global public health, contributing significantly to both premature death and the burden of disability‐adjusted life years (Zhang et al., 2019). In developed countries, the annual death toll from stroke ranges from 50 to 100 per 100,000 individuals (Donnan et al., 2008). This multifaceted health crisis is influenced by a constellation of risk factors, which encompass tobacco smoking, high blood pressure, obesity, high blood cholesterol, hyperlipidemia, coronary heart disease, heavy episodic alcohol consumption, low physical activity, and diabetes (Aigner et al., 2017; Donnan et al., 2008; Zhang et al., 2019). Among these risk factors, tobacco usage stands as a prominent global contributor to premature mortality (Jassem, 2019).

One particular method of tobacco consumption that has gained popularity worldwide is the water pipe, known by various names as hookah, hubble–bubble, narghile, shisha, and qalyan (in Persian) (Montazeri et al., 2017). Its origins trace back approximately 500 years to the regions of Persia (present‐day Iran), Pakistan, and India, from where it gradually diffused across the Middle Eastern (Blachman‐Braun et al., 2014; Montazeri et al., 2017). In recent years, there has been a substantial surge in water pipe use among youth in European, American, African, and Asian (Blachman‐Braun et al., 2014; Momenabadi et al., 2016). Alarmingly, a report from the World Health Organization reveals that the average water pipe smoking session lasts between 20 and 80 min, which is equivalent to or even surpasses the nicotine intake of smoking more than 100 cigarettes a day (Blachman‐Braun et al., 2014; Kim et al., 2016). Remarkably, the volume of smoke generated by water pipe usage is greater that of traditional cigarettes by a factor of 4400–4500 units (Momenabadi et al., 2016).

This context underscores the magnitude of the issue at hand and the need for a comprehensive examination. Notably, the concentration of nicotine in the bloodstream of a daily cigarette smoker is just a fraction of that found in a daily water pipe user (0.1 times) (Kim et al., 2016). Therefore, this study endeavors to present a meta‐analysis of observational studies that delves into the relationship between water pipe smoking and the risk of stroke, an area of research that, though limited, merits attention

## 2 METHODS

We performed the current systematic review and meta‐analysis according to the PRISMA checklist (Moher et al., 2015).

### 2.1 Search strategy

The present study was conducted using the six databases (PubMed, Embase, Web of Science, Ovid SP, Scopus, and Google Scholar) to October 21 2023. The search was not limited to date and language. The following keywords were used to conduct a systematic review and meta‐analysis: stroke [MeSH], “water pipe” [MeSH].

(P) “Tobacco users” OR “Water pipe smokers” [no age limit]

(I) “Hookah use” OR “Water pipe smoking” OR “Narghile” OR “Shisha” OR “water pipe” [MeSH]

(C) “Non‐smokers” OR “Non‐hookah users”

(O) “Stroke incidence” OR “Stroke risk” OR “Stroke severity” OR “Stroke mortality” OR “Stroke disability” OR “Stroke” [MeSH]

### 2.2 Inclusion and exclusion criteria

Studies should meet (1) the observational study (cohort, case‐control, and cross‐sectional), (2) study provided the relationship between water pipe and stroke, and (3) reported the odds ratio (OR) and 95% confidence interval (CI) of relationship between water pipe and stroke, criteria to enter the study.

Exclusion criteria included (1) the stroke patients without a history of water pipe smoking, (2) did not report OR, and (3) irrelevant studies (such as correspondence, letter, review, editorial, ecological, experimental, etc.).

### 2.3 Data extraction

For included studies, the following information was extracted by two authors (DP and BM) independently after reviewing the studies based on the inclusion/exclusion criteria: the first author's name, the publication years, the sample size, the average age and standard deviation of stroke patients, the number of water pipe smokers with a history of stroke, OR and corresponding 95% CI stroke patients based on water pipe smoking, and covariates adjusted for relationship. Any disagreements were resolved by discussion with a third author (HM).

### 2.4 Statistical analysis

The ORs and 95% CIs of stroke in water pipe smokers considered measures of effect size, which was analyzed by the metan package (Harris et al., 2008) in the STATA software version 14.2 (https://www.stata.com) based on standard error and OR from each study. The *Q*‐test and *I* ^2^ statistics (Michael Borenstein & Rothstein, 2007) were used to assess the homogeneity between the studies. According to results from these statistics, we applied the fixed (or random) effect model to obtain the pooled OR for stroke patients who have consumed water pipe. Also, to assess publication bias, funnel plot and Egger's regression test for funnel plot asymmetry were used (Begg & Mazumdar, 1994; Egger et al., 1997). The threshold area to reject results was set as 0.05.

## 3 RESULTS

### 3.1 Study selection

**FIGURE 1:**
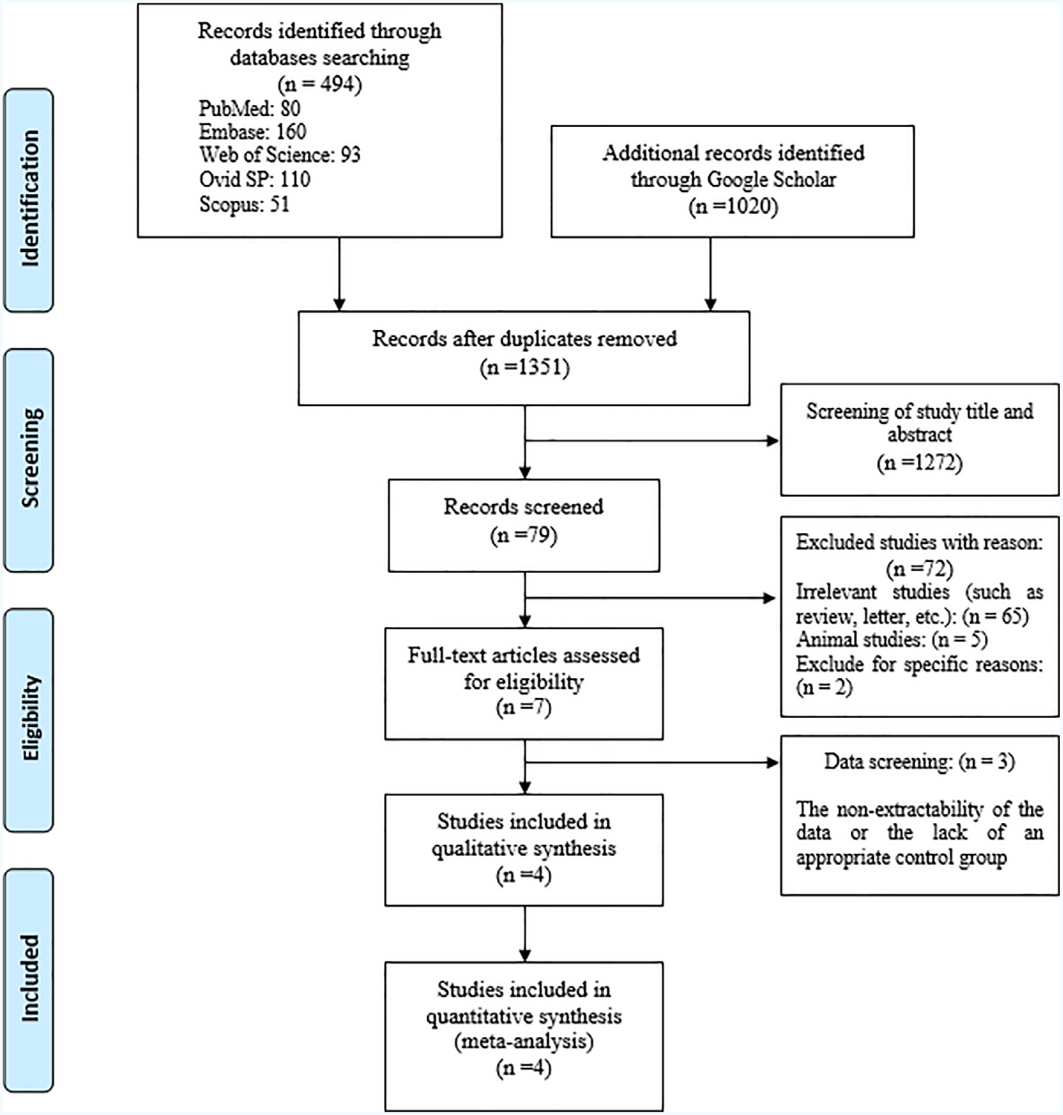
Flow chart for selected eligible studies included in the meta‐analysis.

After implementing the search strategy, a total of 1514 studies were identified. The initial survey showed that 163 studies were duplicated and 1351 studies needed to be further evaluated. A total of 1272 studies were removed after the screening of the study title and abstract. In the next step, 68 excluded for the following reasons: 65 studies were irrelevant or there was no full text, 5 studies were in animal fields, and 2 studies reported the relationship between stroke and water pipe in person year that according to the data presented, OR could not be evaluated. Finally, out of the remaining seven studies, 3 studies were excluded from the study due to the non‐extractability of the data or the lack of an appropriate control group. For the final meta‐analysis, four studies were included (Figure 1).

### 3.2 Characteristics of studies

In the four eligible studies, there were a total of 2759 participants that 555 patients had at least once experienced stroke. Among the include studies, three studies (El‐Hajj et al., 2019, 2020; Tabriz et al., 2020) were case–control studies and one study (Farah et al., 2015) was a cross‐sectional study. The publication year of one study (Farah et al., 2015) was 2015 and the other three studies (El‐Hajj et al., 2019, 2020; Tabriz et al., 2020) were related to the last 2 years. The sample size of four studies ranged from 174 to 1515 participants. In all selected studies, the samples included males and females, but the suggested information was not expressed separately for gender. Also, the information was reported in such a way that the ability to calculate the OR between stroke levels (ischemic, hemorrhagic, and transient) and hookah levels (non‐smoker, former smoker, and current smoker) were not possible. All of the included studies used a logistic model for analysis and adjusted for confounding factors (age, sex, behavior factors, etc.). Further details are present in Table 1.

**TABLE 1:** Characteristics of the included studies.

First Autor name	Country	Study design	Sample size	Stroke subject	Water pipe smokers	Age of stroke subject (mean, SD)	Sex (stroke subject)	Ref.
Rita Farah et al. (2015)	Lebanon	Cross sectional	1515	55	NR	NR	NR	Farah et al. (2015)
Maya El‐Hajj et al. (2019)	Lebanon	Case–control	650	TIA: 31 IS: 143 HS: 31	Non: 106 Current: 83 Former: 16	69.6 ± 12.01	M: 92 F: 113	El‐Hajj et al. (2019)
Reza T et al. (2020)	Iran	Case–control	420	208	56	65.2 ± 15.9	M: 131 F: 77	Tabriz (2020)
Maya El‐Hajj et al. (2020)	Lebanon	Case–control	174	87	Non: 73 Current: 9 Former: 5	72.43 ± 9.63	M: 28 F: 59	El‐Hajj et al. (2020)

Abbreviations: HS, hemorrhagic stroke; IS, ischemic stroke; NR, not reported; SD, standard deviation; TIA, transient ischemic attack.

### 3.3 Water pipe and overall odds of stroke

**FIGURE 2:**
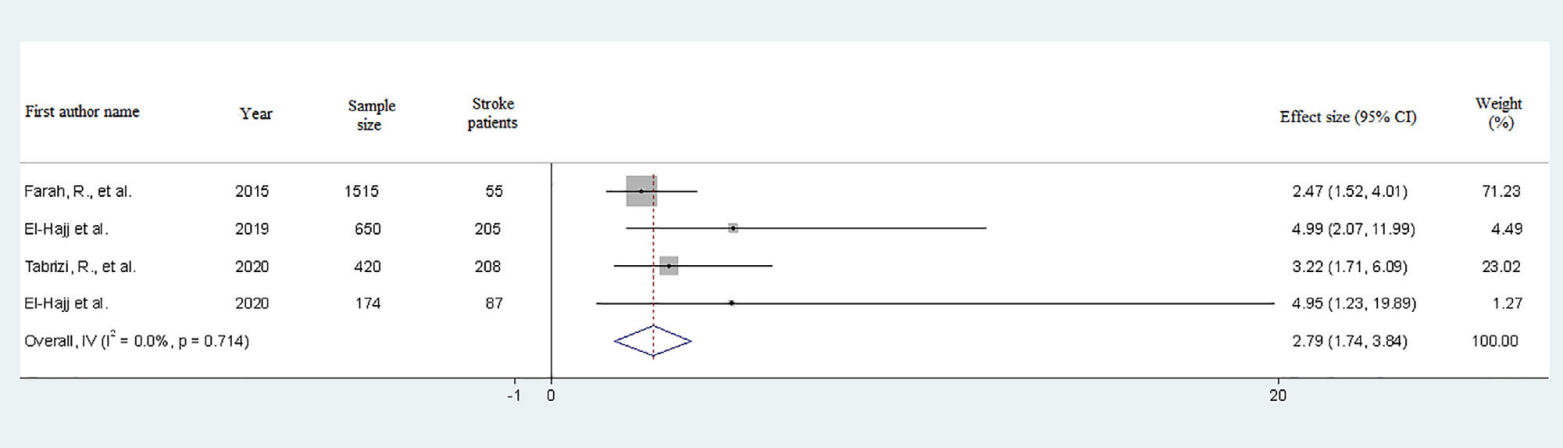
Forest plot of the adjusted relationship between stroke and water pipe smoking.

From the four eligible studies, the result of the meta‐analysis indicated a significant positive relationship between water pipe smoking and stroke (OR = 2.79, 95% CI: 1.74–3.84). Heterogeneity between studies was not statistically significant ($I^{2}\;$= .00%; *p*‐value = .714), and a fixed‐effect model was used in this meta‐analysis (Figure 2).

### 3.4 Publication bias

**FIGURE 3:**
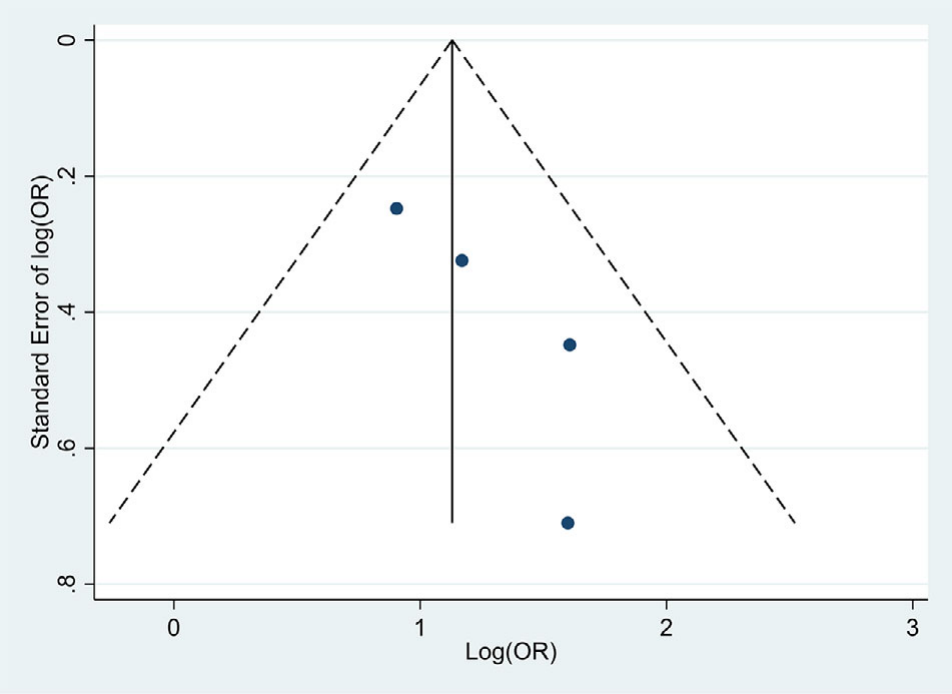
Funnel plot for eligible studies include in meta‐analysis.

To identify the publication bias funnel plot and Egger regression test were used. The funnel plot shows asymmetry publication bias between the four studies (Figure 3). In other words, there was no statistically significant publication bias between studies based on Egger's regression test for funnel plot asymmetry (*t* = 2.70; *p‐*value = .114). These results suggest no publication bias existed in this meta‐analysis.

## 4 DISCUSSION

This meta‐analysis represents the first systematic attempt to explore the association between water pipe smoking and the risk of stroke. Stroke causes 9% of all deaths around the world, which is heavily influenced by various risk factors, including high blood pressure and tobacco use (Donnan et al., 2008). A mere 10 mm Hg increase in systolic blood pressure (or 5 mm Hg in diastolic pressure) can escalated the risk of stroke by 40% (Law et al., 2009). Consequently, high blood pressure is estimated to account for a significant portion, ranging from 35% to 50%, of the overall risk of stroke (Whisnant, 1996). Moreover, smoking leads to a rise in blood pressure, a consequence attributed to the presence of nicotine and carbon monoxide in tobacco smoke (Omvik, 1996).

Carbon monoxide, a component of smoke, binds to blood hemoglobin, forming carboxyhemoglobin, which subsequently reduces blood oxygen levels (Fauci et al., 2012). Evaluated carboxyhemoglobin levels are associated with increased levels of inflammation‐sensitive proteins, such as fibrinogen, haptoglobin, and ceruloplasmin, all of which have established links with cardiovascular disease and stroke. Specifically, heavy smokers with high inflammation‐sensitive protein levels are at a 3.05‐fold higher relative risk of stroke compared to non‐smokers with low inflammation‐sensitive proteins levels (Lind et al., 2004).

Existing knowledge underscores that water pipe use involves substantial levels of nicotine and carbon monoxide, producing a significantly greater volume of smoke in comparison to conventional cigarettes (Sepetdjian et al., 2008). Notwithstanding the rising prevalence of water pipe use and its pronounced adverse effects on physical health in comparison to cigarettes, relatively few meta‐analyses have explored the broad spectrum of health outcome associated with water pipe smoking. Although some have addressed relationships with obesity (Baalbaki et al., 2019; Mamtani et al., 2017), cancer (Chaouachi & Sajid, 2010; Mamtani et al., 2017; Montazeri et al., 2017; Primack et al., 2016; Waziry et al., 2016), low birth weight (Chaouachi & Sajid, 2010; Primack et al., 2016), mental health (Chaouachi & Sajid, 2010; Primack et al., 2016), gastric carcinoma (Chaouachi & Sajid, 2010), cardiovascular disease (Chaouachi & Sajid, 2010; Primack et al., 2016; Raad & al. et al., 2011), mortality (Chaouachi & Sajid, 2010), respiratory diseases (Chaouachi & Sajid, 2010; Primack et al., 2016; Raad & al. et al., 2011), and physical activity (Behrens et al., 2014), no prioir meta‐analysis has ventured into the realm of stroke associated with water pipe use.

The present meta‐analysis, drawing from four eligible studies, reveals a substantial impact of water pipe smoking on the risk of stroke (OR = 2.79, 95% CI: 1.74, 3.84). In each of these studies, individuals who suffered strokes had histories of water pipe and cigarette smoking, hypertension, obesity, and diabetes mellitus. Adjusted OR s in these studies considered factors such as sex, age, and underlying diseases. Furthermore, the absence of heterogeneity and publication bias across these studies lends strength to our findings.

However, it is crucial to acknowledge the limitations of our study. Notably, the available data did not allow for a dose–response analysis, and we did not assess the long‐term effects of water pipe smoking. Additionally, demographic factors, such as age, sex, and education, were not reported in connection to water pipe smoking in some studies. Moreover, in certain instances, cigarette smoking and water pipe smoking were collectively categorized as “smoking,” despite the well‐documented disparities in harm between these two forms of tobacco consumption. Furthermore, there were instances of insufficient detail regarding exposure to water pipe smoke. Finally, all four eligible studies were conducted in the Middle East, which imposes geographical limitations on the scope of our findings. It is essential to recognize that the regional homogeneity of these studies may influence the generalizability of our results.

The popularity of water pipe use, particularly among young individuals, including women, underscores the significant of this issue (Baalbaki et al., 2019). Recent studies indicate a slightly higher prevalence of stroke among women (women: 2.7% and men: 2.6%; about 60% of stroke deaths were attributed to women), and specific factors contributing to strokes in women encompass the use of oral contraceptive pills, pregnancy, preeclampsia, and gestational diabetes among younger women, whereas older women may be at risk due to factors like migraine headaches with aura, atrial fibrillation, diabetes mellitus, and hypertension (Lundberg & Volgman, 2016; Mirzaei, 2017).

In comparing the relationship between cigarette smoking and stroke, previous studies have revealed an increased risk of stroke following cigarette smoking (Fischer & Kraemer, 2015; Lee & Forey, 2006; Lee et al., 2017; Oono et al., 2011; Pan et al., 2019; Shinton & Beevers, 1989; Wilson, 1994). In our meta‐analysis, the OR for water pipe smoking stands at 2.79 (95% CI: 1.74, 3.84), surpassing the OR in meta‐analysis pertaining to cigarette smoking and stroke (current smokers’ odds of stroke compared with non‐smokers was 1.45) (Pan et al., 2019).

## 5 CONCLUSION

In conclusion, our analysis revealed that water pipe smoking has a more substantial effect on stroke risk compared to cigarette smoking, with an OR of 2.79. Given the prevalence of water pipe use, particularly among young people, and considering the high risk it poses, particularly to women, we recommend the implementation of policies aimed at controlling the supply of water pipe products in the market and intensifying educational efforts about the health risks associated with water pipe smoking (Lundberg & Volgman, 2016). Additionally, we encourage researchers to differentiate between cigarettes and water pipe smoking in their analyses, and in light of the increasing popularity of water pipes, we advocate for further comprehensive studies to investigate the relationship between water pipe use and other diseases, especially stroke.

The limitations and regional focus of our study should be acknowledged, and future research should aim to expand the geographical and demographic diversity of the studies, providing a more comprehensive understanding of the relationship between water pipe smoking and stroke.

## AUTHOR CONTRIBUTIONS

**Mehrdad Bagherpour‐Kalo**: Conceptualization; data curation; formal analysis; funding acquisition; investigation; methodology; project administration; resources; software; supervision; validation; visualization; writing—original draft; writing—review and editing. **Michael E Jones**: Investigation; methodology; validation; writing—original draft. **Parvaneh Darabi**: Data curation; software; writing—original draft. **Mostafa Hosseini**: Conceptualization; investigation; methodology; project administration; writing—original draft; writing—review and editing.

## CONFLICT OF INTERESTS STATEMENT

All authors declare there are no conflicts of interest.

## FUNDING INFORMATION

None

### PEER REVIEW

The peer review history for this article is available at https://publons.com/publon/10.1002/brb3.3357.

## Data Availability

The datasets used in current study is available in context of study.

## References

[brb33357-bib-0001] Aigner, A. , Grittner, U. , Rolfs, A. , Norrving, B. , Siegerink, B. , & Busch, M. A. (2017). Contribution of established stroke risk factors to the burden of stroke in young adults. Stroke; A Journal of Cerebral Circulation, 48(7), 1744–1751. 10.1161/STROKEAHA.117.016599 28619986

[brb33357-bib-0002] Baalbaki, R. , Itani, L. , El Kebbi, L. , Dehni, R. , Abbas, N. , Farsakouri, R. , Awad, D. , Tannir, H. , Kreidieh, D. , El Masri, D. , & El Ghoch, M. (2019). Association between smoking hookahs (shishas) and higher risk of obesity: A systematic review of population‐based studies. Journal of Cardiovascular Development and Disease, 6(1), 23. 31208138 10.3390/jcdd6020023 PMC6617155

[brb33357-bib-0003] Begg, C. B. , & Mazumdar, M. (1994). Operating characteristics of a rank correlation test for publication bias. Biometrics, 50(4), 1088–1101. 10.2307/2533446 7786990

[brb33357-bib-0004] Behrens, G. , Jochem, C. , Keimling, M. , Ricci, C. , Schmid, D. , & Leitzmann, M. F. (2014). The association between physical activity and gastroesophageal cancer: Systematic review and meta‐analysis. European Journal of Epidemiology, 29(3), 151–170. 10.1007/s10654-014-9895-2 24705782

[brb33357-bib-0005] Blachman‐Braun, R. , Del Mazo‐Rodríguez, R. L. , López‐Sámano, G. , & Buendía‐Roldán, I. (2014). Hookah, is it really harmless? Respiratory Medicine, 108(5), 661–667. 10.1016/j.rmed.2014.01.013 24582881

[brb33357-bib-0006] Chaouachi, K. , & Sajid, K. M. (2010). A critique of recent hypotheses on oral (and lung) cancer induced by water pipe (hookah, shisha, narghile) tobacco smoking. Medical Hypotheses, 74(5), 843–846. 10.1016/j.mehy.2009.11.036 20036075

[brb33357-bib-0007] Donnan, G. A. , Fisher, M. , Macleod, M. , & Davis, S. M. (2008). Stroke. The Lancet, 371(9624), 1612–1623. 10.1016/S0140-6736(08)60694-7 18468545

[brb33357-bib-0008] Egger, M. , Smith, G. D. , Schneider, M. , & Minder, C. (1997). Bias in meta‐analysis detected by a simple, graphical test. BMJ, 315(7109), 629–634. 10.1136/bmj.315.7109.629 9310563 PMC2127453

[brb33357-bib-0009] El‐Hajj, M. , Salameh, P. , Rachidi, S. , Al‐Hajje, A. , & Hosseini, H. (2019). Cigarette and water pipe smoking are associated with the risk of stroke in Lebanon. Journal of Epidemiology and Global Health, 9(1), 62–70. 10.2991/jegh.k.181231.002 30932392 PMC7310762

[brb33357-bib-0010] Farah, R. , Zeidan, R. K. , Chahine, M. N. , Asmar, R. , Chahine, R. , Salameh, P. , & Hosseini, H. (2015). Prevalence of stroke symptoms among stroke‐free residents: First national data from Lebanon. International Journal of Stroke, 10(Suppl A100), 83–88. 10.1111/ijs.12563 26178607

[brb33357-bib-0011] Fischer, F. , & Kraemer, A. (2015). Meta‐analysis of the association between second‐hand smoke exposure and ischaemic heart diseases, COPD and stroke. BMC Public Health, 15, 1202. 26627181 10.1186/s12889-015-2489-4 PMC4667413

[brb33357-bib-0012] Hajj, M. , Ajrouche, R. , Zein, S. , Rachidi, S. , Awada, S. , & Al‐Hajje, A. (2020). Evaluation of risk factors and drug adherence in the occurrence of stroke in patients with atrial fibrillation. Pharmacy Practice, 18(2), 1860–1860. 10.18549/PharmPract.2020.2.1860 32566048 PMC7290178

[brb33357-bib-0013] Harris, R. , Deeks, J. J. , Altman, D. G. , Bradburn, M. J. , Harbord, R. M. , & Sterne, J. A. C. (2008). Metan: Fixed‐ and random‐effects meta‐analysis. The Stata Journal: Promoting Communications on Statistics and Stata, 8, 3–28.

[brb33357-bib-0014] Jassem, J. (2019). Tobacco smoking after diagnosis of cancer: Clinical aspects. Translational Lung Cancer Research, 8(Suppl1), S50–S58. 10.21037/tlcr.2019.04.01 31211105 PMC6546630

[brb33357-bib-0015] Kim, K.‐H. , Kabir, E. , & Jahan, S. A. (2016). Waterpipe tobacco smoking and its human health impacts. Journal of Hazardous Materials, 317, 229–236. 10.1016/j.jhazmat.2016.05.075 27285594

[brb33357-bib-0016] La Fauci, G. , Weiser, G. , Steiner, I. P. , & Shavit, I. (2012). Carbon monoxide poisoning in narghile (water pipe) tobacco smokers. Canadian Journal of Emergency Medicine, 14(1), 57–59. 22417961 10.2310/8000.2011.110431

[brb33357-bib-0017] Law, M. R. , Morris, J. K. , & Wald, N. J. (2009). Use of blood pressure lowering drugs in the prevention of cardiovascular disease: Meta‐analysis of 147 randomised trials in the context of expectations from prospective epidemiological studies. BMJ, 338, b1665. 10.1136/bmj.b1665 19454737 PMC2684577

[brb33357-bib-0018] Lee, P. N. , & Forey, B. A. (2006). Environmental tobacco smoke exposure and risk of stroke in nonsmokers: A review with meta‐analysis. Journal of Cardiovascular Development and Disease, 15(5), 190–201. 10.1016/j.jstrokecerebrovasdis.2006.05.002 17904075

[brb33357-bib-0019] Lee, P. N. , Thornton, A. J. , Forey, B. A. , & Hamling, J. S. (2017). Environmental tobacco smoke exposure and risk of stroke in never smokers: An updated review with meta‐analysis. Journal of Stroke and Cerebrovascular Diseases, 26(1), 204–216. 10.1016/j.jstrokecerebrovasdis.2016.09.011 27765554

[brb33357-bib-0020] Lind, P. , EngströM, G. , Stavenow, L. , Janzon, L. , LindgäRde, F. , & Hedblad, B. (2004). Risk of myocardial infarction and stroke in smokers is related to plasma levels of inflammation‐sensitive proteins. Arteriosclerosis, Thrombosis, and Vascular Biology, 24(3), 577–582. 10.1161/01.ATV.0000116863.37311.82 14726408

[brb33357-bib-0021] Lundberg, G. P. , & Volgman, A. S. (2016). Burden of stroke in women. Trends in Cardiovascular Medicine, 26(1), 81–88. 10.1016/j.tcm.2015.04.010 26051206

[brb33357-bib-0022] Mamtani, R. , Cheema, S. , Sheikh, J. , Al Mulla, A. , Lowenfels, A. , & Maisonneuve, P. (2017). Cancer risk in waterpipe smokers: A meta‐analysis. International Journal of Public Health, 62(1), 73–83. 10.1007/s00038-016-0856-2 27421466 PMC5288449

[brb33357-bib-0023] Borenstein, M. , Hedges, L. , & Rothstein, H. (2007). Meta‐analysis fixed effect vs. random effects. Comprehensive Meta‐Analysis. www.Meta‐Analysis.com 10.1002/jrsm.12 26061376

[brb33357-bib-0024] Mirzaei, H. (2017). Stroke in women: Risk factors and clinical biomarkers. Journal of Cellular Biochemistry, 118(12), 4191–4202. 10.1002/jcb.26130 28498508

[brb33357-bib-0025] Moher, D. , Shamseer, L. , Clarke, M. , Ghersi, D. , Liberati, A. , Petticrew, M. , Shekelle, P. , & Stewart, L. A. (2015). Preferred reporting items for systematic review and meta‐analysis protocols (PRISMA‐P) 2015 statement. Systematic Reviews, 4(1), 1. 10.1186/2046-4053-4-1 25554246 PMC4320440

[brb33357-bib-0026] Momenabadi, V. , Hossein Kaveh, M. , Hashemi, S. Y. , & Borhaninejad, V. R. (2016). Factors affecting hookah smoking trend in the society: A review article. Addiction & Health, 8(2), 123–135. 27882210 PMC5115646

[brb33357-bib-0027] Montazeri, Z. , Nyiraneza, C. , El‐Katerji, H. , & Little, J. (2017). Waterpipe smoking and cancer: Systematic review and meta‐analysis. Tobacco Control, 26(1), 92–97. 10.1136/tobaccocontrol-2015-052758 27165994

[brb33357-bib-0028] Omvik, P. (1996). How smoking affects blood pressure. Blood Pressure, 5(2), 71–77. 9162447 10.3109/08037059609062111

[brb33357-bib-0029] Oono, I. P. , Mackay, D. F. , & Pell, J. P. (2011). Meta‐analysis of the association between secondhand smoke exposure and stroke. Journal of Public Health (Oxford, England), 33(4), 496–502. 10.1093/pubmed/fdr025 21422014

[brb33357-bib-0030] Pan, B. , Jin, X. , Jun, L. , Qiu, S. , Zheng, Q. , & Pan, M. (2019). The relationship between smoking and stroke: A meta‐analysis. Medicine, 98(12), e14872. 10.1097/MD.0000000000014872 30896633 PMC6708836

[brb33357-bib-0031] Primack, B. A. , Carroll, M. V. , Weiss, P. M. , Shihadeh, A. L. , Shensa, A. , Farley, S. T. , Fine, M. J. , Eissenberg, T. , & Nayak, S. (2016). Systematic review and meta‐analysis of inhaled toxicants from water pipe and cigarette smoking. Public Health Reports, 131(1), 76–85. 10.1177/003335491613100114 26843673 PMC4716475

[brb33357-bib-0032] Raad, D. , Gaddam, S. , Schunemann, H. J. , Irani, J. , Abou Jaoude, P. , Honeine, R. , & Akl, E. A. (2011). Effects of water‐pipe smoking on lung function: A systematic review and meta‐analysis. Chest, 139(4), 764–774. 10.1378/chest.10-0991 20671057

[brb33357-bib-0033] Sepetdjian, E. , Shihadeh, A. , & Saliba, N. A. (2008). Measurement of 16 polycyclic aromatic hydrocarbons in narghile waterpipe tobacco smoke. Food and Chemical Toxicology, 46(5), 1582–1590. 10.1016/j.fct.2007.12.028 18308445

[brb33357-bib-0034] Shinton, R. , & Beevers, G. (1989). Meta‐analysis of relation between cigarette smoking and stroke. BMJ, 298(6676), 789–794. 10.1136/bmj.298.6676.789 2496858 PMC1836102

[brb33357-bib-0035] Tabrizi, R. , Borhani‐Haghighi, A. , Lankarani, K. B. , Heydari, S. T. , Bayat, M. , Vakili, S. , Maharlouei, N. , Hassanzadeh, J. , Zafarmand, S. S. , Owjfard, M. , Avan, A. , & Azarpazhooh, M. R (2020). Hookah smoking: A potentially risk factor for first‐ever ischemic stroke. Journal of Stroke and Cerebrovascular Diseases, 29(10), 105138. 10.1016/j.jstrokecerebrovasdis.2020.105138 32912523

[brb33357-bib-0036] Waziry, R. , Jawad, M. , Ballout, R. A. , Al Akel, M. , & Akl, E. A. (2016). The effects of waterpipe tobacco smoking on health outcomes: An updated systematic review and meta‐analysis. International Journal of Epidemiology, 46(1), 32–43. 10.1093/ije/dyw021 27075769

[brb33357-bib-0037] Whisnant, J. P. (1996). Effectiveness versus efficacy of treatment of hypertension for stroke prevention. Neurology, 46(2), 301–307. 10.1212/WNL.46.2.301 8614485

[brb33357-bib-0038] Wilson, E. (1994). Enhancing smoke‐free behaviour: Prevention of stroke. Health Reports, 6(1), 100–105. 7919064

[brb33357-bib-0039] Zhang, S. , Zhang, W. , & Zhou, G. (2019). Extended risk factors for stroke prevention. Journal of the National Medical Association, 111(4), 447–456. 30878142 10.1016/j.jnma.2019.02.004

